# Identification of a *Bacillus thuringiensis *Cry11Ba toxin-binding aminopeptidase from the mosquito, *Anopheles quadrimaculatus*

**DOI:** 10.1186/1471-2091-7-16

**Published:** 2006-05-22

**Authors:** Mohd Amir F Abdullah, Algimantas P Valaitis, Donald H Dean

**Affiliations:** 1 Department of Biochemistry and The Protein Research Group, The Ohio State University, Columbus, Ohio 43210, USA; 2 Department of Science, Faculty of Engineering, International Islamic University Malaysia, Kuala Lumpur, Malaysia; 3 Department of Entomology, University of Georgia, Athens, GA 30602, USA; 4 USDA Forest Service, Delaware, Ohio, USA

## Abstract

**Background:**

Aminopeptidase N (APN) type proteins isolated from several species of lepidopteran insects have been implicated as *Bacillus thuringiensis *(Bt) toxin-binding proteins (receptors) for Cry toxins. We examined brush border membrane vesicle (BBMV) proteins from the mosquito *Anopheles quadrimaculatus *to determine if APNs from this organism would bind mosquitocidal Cry toxins that are active to it.

**Results:**

A 100-kDa protein with APN activity (APN_Anq _100) was isolated from the brush border membrane of *Anopheles quadrimaculatus*. Native state binding analysis by surface plasmon resonance shows that APN_Anq _100 forms tight binding to a mosquitocidal Bt toxin, Cry11Ba, but not to Cry2Aa, Cry4Ba or Cry11Aa.

**Conclusion:**

An aminopeptidase from *Anopheles quadrimaculatus *mosquitoes is a specific binding protein for *Bacillus thuringiensis *Cry11Ba.

## Background

The main African vectors of malaria are in the *Anopheles gambiae *complex mosquitoes [1]. In general, all species of *Anopheles *have been found to be susceptible to a certain extent to infection by some strain of human plasmodia [2]. Studies on lepidopteran insects revealed several types of Bt toxin-binding proteins (receptors): aminopeptidase N (APN) -like proteins [3,4]; cadherin-like proteins [5,6]; a glycoconjugate [7] and glycolipids [8]. In mosquitoes, two types of receptors were discovered: a protein with maltase activity from *Culex. pipiens *that binds the Bin toxin of *Bacillus sphaericus *[9], and a 65 kDa protein of unknown function (lacking aminopeptidase activity) from *Aedes aegypti *that binds Cry4Ba and Cry11Aa [10]. Two APNs have been identified in *Ae. aegypti *but not associated with binding Cry proteins [11]

APNs (EC 3.4.11.2) are exopeptidases that cleave single amino acids from the N-terminus of a polypeptide. APNs are expressed in many tissues including the brain, the lung, blood vessels, primary cultures of fibroblasts [12], and have the highest levels in intestinal and kidney brushborder membranes [13]. APNs belong to the M_1 _family of zinc metallopeptidases [14], which includes related enzymes like aminopeptidase A [15], aminopeptidase B [16,17], and leukotriene A4 hydrolase [18]. APNs have also been implicated as cellular receptors for human, canine, and feline coronaviruses [19].

In this study, intestinal APN from *An. quadrimaculatus *larvae was isolated and tested for binding ability to different mosquitocidal Cry toxins (Cry2Aa, Cry4Ba, Cry11Aa, and Cry11Ba). Membrane proteins were extracted from *An. quadrimaculatus *brush border membrane vesicles (BBMV) and separated by anion-exchange chromatography. Fractions containing APN activity were pooled and purified by size-exclusion chromatography. A 100-kDa protein with APN activity was isolated from the BBMV and its N-terminal sequence was determined to be AQLEDYRLNDDVRPTAYRIE. This protein was used to screen different mosquitocidal Cry toxins binding via Biacore analysis. From the screening, it was discovered that only Cry11Ba was able to bind the APN. A protein BLAST search limited to the arthropod database revealed three highly homologous *An. gambiae *APNs based on the N-terminal sequence.

## Results

### Purification of An. quadrimaculatus aminopeptidase N

**Figure 1:**
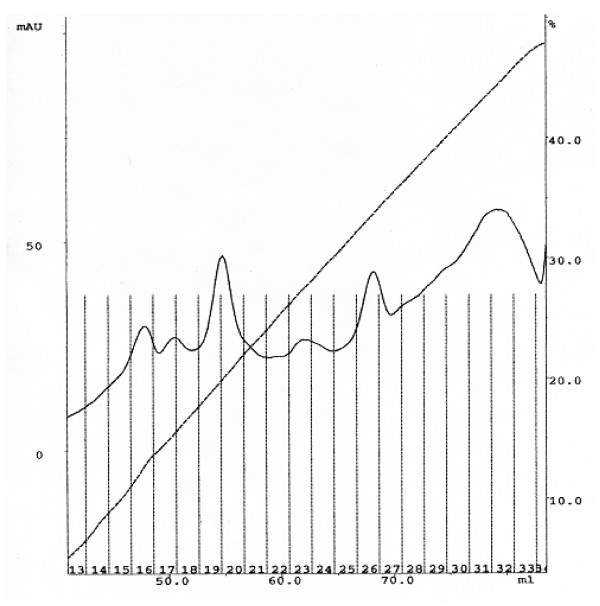
Separation of *An. quadrimaculatus *aminopeptidase N from solubilized BBMV proteins by anion-exchange chromatography. The UV absorbance at 280 nm (mAU) is indicated at the top left corner, and the percent conductivity of buffer B (%) is indicated at the top right corner. Collected fractions are shown at the bottom in 2-ml intervals. Run volume is indicated at the bottom (ml). Fractions 19–21 and 24–34 contain APN activity.

**Figure 2:**
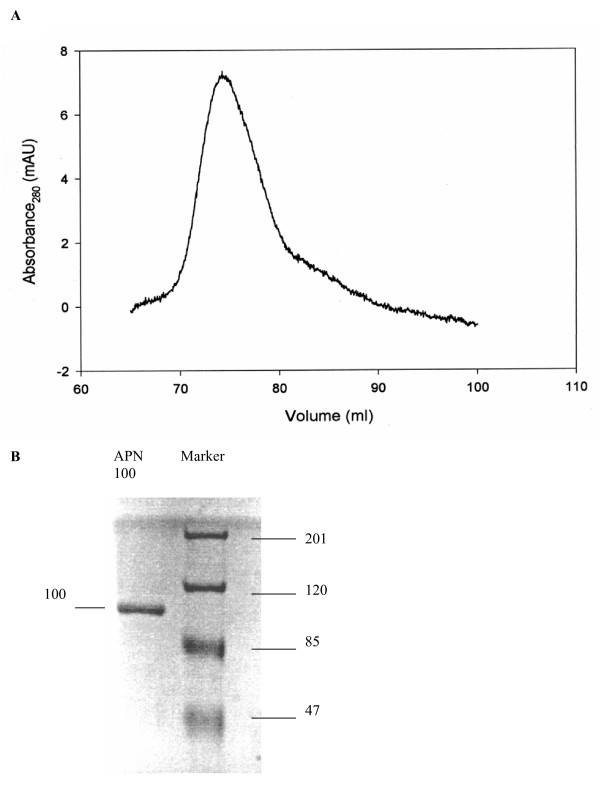
(**A**) Further purification of APN fractions (fractions 19–21) from anion-exchange chromatography of *An. quadrimaculatus *BBMV by size-exclusion chromatography. A single peak was eluted at 75 ml elution volume, corresponding to 100 kDa. (**B**) SDS-PAGE of purified APN (APN_Anq _100) obtained in (**A**) above. The estimated sizes of the protein bands are indicated on both sides of the gel in kDa.

SPR analysis requires purified ligands and analytes to be used. Solubilized *An. quadrimaculatus *BBMV proteins were separated by anion-exchange chromatography and all elution fractions were tested for APN activity. Fractions 19–21 and 24–34 showed APN activity. Fractions 19–21 were made up of a single peak, and fractions 24–34 were made up of at least two peaks (Fig. 1). Fractions 19–21 were pooled, concentrated, and purified further by size-exclusion chromatography. A single peak was eluted at around 75 ml of run volume that correspond to a protein size of about 100 kDa (Fig. 2A). This peak was collected and was determined to hold APN activity. SDS-PAGE analysis of the protein also indicated a size of 100 kDa (Fig. 2B) and the 100 kDa protein was highly purified. The 100 kDa protein was named APN_Anq _100.

### Determination of binding affinity by SPR analysis

**Figure 3:**
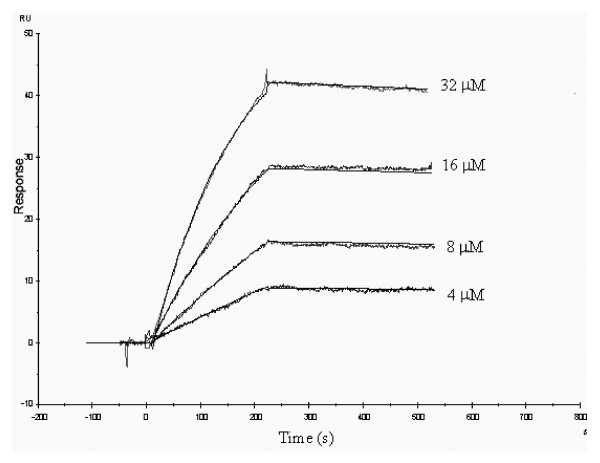
Real-time binding of Cry11Ba to *An. quadrimaculatus *APN_Anq _100. Experimental curves (jagged line) are shown overlaid with fitted curves (smooth line) obtained with the 1:1 Langmuir binding with drifting baseline model. The overlaid BIAcore response curves are shown for Cry11Ba toxin injections at 4, 8, 16, 32 μM as indicated.

Initially, APN_Anq _100 was evaluated for binding by SPR analysis to four Cry toxins (Cry2Aa, Cry4Ba, Cry11Aa and Cry11Ba), which were previously determined in this laboratory to have mosquitocidal activity towards *An. quadrimaculatus *(data not shown). Only Cry11Ba bound significantly to APN_Anq _100. Further analysis of real-time binding kinetic of Cry11Ba to APN_Anq _100 was performed at different analyte concentrations (Fig. 3), followed by global fitting of all the response curves. A 1:1 binding stoichiometry, including a drifting-baseline correction, produced the following apparent rate constants of the bimolecular interaction: *k*_*a *_= 184.0 M^-1^s^-1 ^(± 1.0) and *k*_*d *_= 1.03 × 10^-7 ^s^-1 ^(± 4.01 × 10^-6^), K_*D *_= 0.56 nM. More complex binding models, such as 2-site independent binding (A + B1 ↔ AB1; A + B2 ↔ AB2), and 2-site sequential binding (A + B ↔ AB ↔ AB*) also gave as good fitting as the simple 1:1 binding (A + B ↔ AB) with χ^2 ^= 0.112 (data not shown).

### N-terminal sequence of APNAnq100

A twenty amino acid residue sequence (AQLEDYRLNDDVRPTAYRIE) was obtained from N-terminal sequencing of purified APN_Anq _100. Data mining for similar sequences in the arthropod databases revealed high homology (80–85% identities) with 3 conceptual translated proteins from *An. gambiae *(Table 1). A BLAST search using the first protein's full amino acid sequence from *An. gambiae *(accession no. EAA08760.1) revealed homology with many aminopeptidases from organisms of other genera (data not shown). This would suggest that the three proteins from *An. gambiae *have aminopeptidase activity.

**Table 1:** Amino acid sequence similarities of the N-terminal sequence of APN_Anq _100 from *An. quadrimaculatus *with three protein sequences from *An. gambiae *obtained through a BLAST search.

Source identity Acc. No.^b^	Amino acid sequence^a^	% identity
*An. quadrimaculatus*	1-AQLEDYRLNDDVRPTAYRIE-20	NA^c^
*An. gambiae *EAA08760.1	42-AQLEDYRLNDDVWPTHYDIE-61	85
*An. gambiae *EAA08929.1	53-AQLEEYRLNDDVWPTHYDIE-72	85
*An. gambiae *EAA08763.1	45-AQPEDYRLNDDVWPTHYDIE-64	80

^a ^The numbers flanking the sequences represent residue position in the protein.

^b ^The accession no. in protein database.

^c ^NA- Not applicable.

Analysis of the N-terminal region with the program SignalP () predicted that the most probable cleavage site for the signal peptide sequence was between position 25 and 26 for EAA08760.1; between position 27 and 28 for EAA08763.1; and between position 28 and 29 for EAA08929.1. However, the sequences of the proteins shown in Table 2 start at positions further downstream from the predicted cleavage sites, which suggested that there might have been further processing of the N-terminal region of the *An. quadrimaculatus *APN. Analysis of the C-terminal region for possible glycosylphosphatidylinositol (GPI) anchor sites using the program Big-PI Predictor () found no potential GPI-modification site for EAA08760.1. Potential GPI-modification sites were found at position 930 and 920 for EAA08763.1 and EAA08929.1, respectively. Analysis of the sequences using the program NetOGlyc 2.0 () [20] to reveal potential GalNAc O-glycosylation sites found 5 sites in EAA08760.1, 7 sites in EAA08763.1, and 6 sites in EAA08929.1. Analysis of the sequences using NetNGlyc 1.0 () [21] to reveal potential N-glycosylation sites found 2 sites in EAA08760.1, 8 sites in EAA08763.1, and 3 sites in EAA08929.1.

**Table 2:** Putative aminopeptidases in *An. gambiae *that contain a conserved MAAVPDFSAGAMENWGLL sequence.

No.	Accession no.	Protein length (residues)
1	EAA05382.1	649
2	EAA01063.1	1800
3	EAA13235.1	1691
4	EAA09719.1	734
5	EAA08912.1	811
6	EAA02981.1	641
7	EAA08915.1	870
8	EAA08931.1	997
9	EAA12046.1	955
10	EAA10722.1*	809
11	EAA08434.1	990
12	EAA08760.1	791
13	EAA08910.1	614
14	EAA08929.1	940
15	EAA03210.1	639
16	EAA08763.1	952

* HEXXH motif for the APN zinc-iron-binding site does not exist in this sequence, which would exclude this protein from the metallopeptidase family.

Another protein BLAST search was performed using the sequence of a known conserved region for aminopeptidases (MAAVPDFSAGAMENWGLL) [22], which yielded 16 homologous proteins from the *An. gambiae *genomic database (Table 2). This indicated that there are a large number of aminopeptidase isomers in these mosquitoes.

## Discussion and conclusion

An aminopeptidase N (APN) type protein has been implicated as a Cry toxin-binding protein in several lepidopteran species: *Manduca sexta *[4], *Bombyx mori *[23,24], *Lymantria dispar *[25,26], *Heliothis virescens *[27], *Plutella xylostella *[28], *Trichoplusia ni *[29], *Helicoverpa armigera *[30] and *Spodoptera litura *[31]. Recently the binding epitopes of Cry1Aa to an APN from *B. mori *have been mapped by monoclonal antibody inhibition [32]. Thus, targeting APN for analysis as a possible toxin-binding protein is a reasonable approach.

The surface plasmon resonance (SPR) method allows analysis of bimolecular interaction in the native state, without a potentially interfering label [33]. Thus, since the Cry11Ba and APN_Anq _100 interaction detected in this study represents tight (ca. 1 nM K_*D *_) native-state binding, we propose that APN_Anq _100 is a putative receptor for Cry11Ba. APN_Anq _100 did not bind to Cry2Aa, Cry4Ba or Cry11Aa even though the toxins have insecticidal activity against *An. quadrimaculatus*. The specific binding of Cry11Ba to APN_Anq _100 suggests that its mode of action would be different from Cry2Aa, Cry4Ba, or Cry11Aa.

The N-terminal sequence of APN_Anq _100 showed high homology with three putative APNs from *An. gambiae*. One or more of these APNs could act as a binding protein for Cry11Ba.

Recently the binding epitopes of Cry1Aa to an APN from *B. mori *have been mapped by monoclonal antibody inhibition [32].

## Methods

### Preparation of mosquito brush border membrane vesicles (BBMV)

Fourth instars *An. quadrimaculatus *larvae were filtered with a nylon mesh, washed in distilled water, separated from large residual food particles, and dried briefly on a filter paper (Fisher) under vacuum suction. Harvested larvae were frozen at -70°C until needed. About 4–6 g of frozen larvae were homogenized in 8–12 ml of cold buffer A (300 mM mannitol, 5 mM EGTA, 17 mM Tris-HCl, pH 7.5). Larvae were homogenized by 40 strokes of Potter-Elvehjem PTFE pestle in glass tube at speed number 5 (~6000 rpm). BBMV were enriched through differential centrifugation by selective divalent-cation precipitations as described by Silva-Filha, et al [34]. The BBMV pellet was resuspended in 1 ml of ice-cold binding buffer (8 mM NaHPO_4 _, 2 mM KH_2 _PO_4 _, 150 mM NaCl, pH 7.4) supplemented with COMPLETE™ (Roche) protease inhibitor and homogenized by10 extrusions using a small Teflon pestle.The protein concentration of the BBMV was measured with the Coomassie protein assay reagent (Pierce), using BSA as the standard. The BBMV was kept at -70°C until needed.

### Purification of An. quadrimaculatus aminopeptidase N (APN) from BBMV

Approximately 20 mg of BBMV was solubilized overnight at 4°C in the binding buffer supplemented with 10 mg/ml of CHAPS (Roche). Later, the solution was vortexed briefly and centrifuged at 15,000 rpm in a JA-17 rotor at 4°C for 10 min. The supernatant was treated with PIPLC for 1 hr at 37°C. The supernatant was separated by anion-exchange chromatography (HiTrap 5 ml column, Pharmacia) by continuous salt gradient using two buffers: A, 20 mM Tris-Cl, pH 7.4, 0.4 mg/ml CHAPS; B, buffer A with 1 M NaCl. Two milliliters elution fractions were collected at a flow rate of 1 ml/min. A small fraction of each elution fraction was tested for the presence of APN activity using L-leucine *p*-nitroanilide (Sigma) as substrate. Neighboring fractions containing APN activities were pooled and concentrated using centricon (YM30, Millipore) according to the manufacturer. The pooled fractions were further purified by size exclusion chromatography (Superdex 200, Pharmacia) in 20 mM Tris, pH 7.4, 0.4 mg/ml CHAPS and concentrated as before. The quality of the sample was checked by sodium dodecyl sulfate-polyacrylamide gel electrophoresis (SDS-PAGE) as described by Laemmli [35].

### Purification of Cry toxins

An *E. coli *clone of Cry2Aa (a grateful gift from Takashi Yamamoto) was used as a source of this gene. The *cry2Aa *gene was extracted by PCR and cloned into plasmid pHT600 and transformed into *B. thuringiensis *4Q7, a plasmidless Cry^- ^derivitive. The genes *cry4Aa*, *cry4Ba*, *cry11Aa *and *cry11Ba *were received in the same plasmid vector and host *B. thuringiensis *strain (gratefully donated by Armelle Delécluse). Single Bt colonies were inoculated into a 5 ml LB medium supplemented with 10 μg/ml erythromycin and grown overnight at 30°C in an incubator-shaker at 250 rpm. These cultures were inoculated into a 500 ml SSM medium [36] also supplemented with erythromycin and incubated a further 4 days until sporulation and autolysis. Bt crystals in the autolysed-cells suspension were purified as described previously [37] for purification of Cry toxins expressed in *E. coli*, except that the sonication steps were omitted. The crystals were solubilized in carbonate buffer (30 mM Na_2 _CO_3 _, 20 mM NaHCO_3 _, pH 10.0) supplemented with 10 mM dithiothreitol (Roche) at 37°C for 3 hours. Next, the solubilized toxin was incubated with 1/20 (v/v) 10 mg/ml trypsin (Sigma) at 37°C for 3 hours. The activated toxin was purified by FPLC using a Superdex 200 (Pharmacia) column in the carbonate buffer. Protein concentration was measured using the Coomassie protein assay reagent (Pierce) with bovine serum albumin as standard.

### Biosensor analysis of toxin-APN affinities

All surface plasmon resonance (SPR) experiments were performed on a BIAcore 3000 machine (Biacore AB). *An. quadrimaculatus *APN in 20 mM ammonium acetate, pH 4.2, was immobilized on a CM5 sensor chip by amine-coupling method (Biacore AB). The flow buffer HBS-EP (10 mM HEPES, 150 mM NaCl, 3 mM EDTA, 0.005% polysorbate 20 (v/v), pH 7.4) (Biacore AB) was used at a flow rate of 30 μl/min. Multiple concentrations (4, 8, 16, and 32 μM) of Cry11Ba was injected across the flow cell containing the APN and one blank flow cell containing ethanolamine as a blocking agent. Surfaces were regenerated with 2 pulses of 10 μl of 10 mM NaOH, pH 11, at 100 μl/min or until the signal return to baseline. Signal responses from the blank flow cells were subtracted from all response curves and data were globally fitted using BIAevaluation Ver. 3.1 (Biacore AB). The curves were fitted to a simple 1:1 Langmuir binding model (A+B ↔ AB) to obtain apparent rate constants.

### N-terminal sequencing and sequence similarity search

For N-terminal sequencing, proteins separated in SDS-PAGE were transferred onto PVDF membrane (Roche) by electro-transfer (Mini-PROTEAN™ II, Bio Rad) according to the manufacturer. The membrane was stained briefly with Coomassie Blue R-250 and destained in 50% methanol. Bands representing 100-kDa proteins were excised and sequencing was performed on an automated sequencer (Model 477A, Applied Biosystems) at USDA Forest Service Laboratory, Delaware, OH. Data mining was performed on the N-terminal sequence using the basic local alignment search tool (BLAST), an on-line tool, at the National Center for Biotechnology Information (NCBI) website. The search parameter was limited to arthropods. CLUSTAL W () was used to align the amino acid sequences.

## Authors' contributions

MAFA and DHD planned the study and wrote the initial draft of the manuscript. MAFA conducted all experiments, except the N-terminal amino acid analysis. APV conducted the N-terminal amino acid analysis. All authors were involved in revising the manuscript and giving final approval of the version to be published.
